# A comparison of work characteristics and health status between Korean and US hospital nurses

**DOI:** 10.1002/nop2.70040

**Published:** 2024-09-17

**Authors:** Kihye Han, Alison M. Trinkoff, Hyang Baek, Yeonhee Kim

**Affiliations:** ^1^ Chung‐Ang University College of Nursing Seoul South Korea; ^2^ University of Maryland School of Nursing Baltimore Maryland USA; ^3^ University of Ulsan Department of Clinical Nursing Seoul South Korea

**Keywords:** health, Korea, nurse, organizational support, United States, work conditions

## Abstract

**Aim:**

A rigorous examination of the occupational features across cultures helps draw policy recommendations for nurses' quality care practices and good health. This study aimed to explore the differences in work characteristics and health status between Korean and US hospital nurses.

**Design:**

For this comparative secondary data analysis study, we constructed a dataset with 304 pairs of nurses from Korea and the United States, matched by age and gender.

**Methods:**

We used the data from the 2020 Korean Hospital Nurses Health Behaviors and Health Status study, collected from May to July 2020, and the Nurse Worklife and Wellness Study (NWWS), conducted between November 2020 and February 2021.

**Results:**

Compared to nurses in the United States, Korean nurses rated their job‐related conditions much lower, had lower intentions to stay in their current workplace and were less satisfied with their jobs. Korean nurses reported that organizational support and employee health resources were less prevalent and their levels of healthy behaviour practice and health status were lower than their US counterparts. Nurses in Korea require better practice environments and employee health support. Adequate workload and staffing levels are needed to improve job conditions for Korean nurses. Organizational support and employee health resources should always be accessible at nurses' workplaces.

## 1 INTRODUCTION

Nurses are key personnel in hospital settings who provide 24/7 patient care. The practice environmental characteristics and nurses' health status are directly linked to their ability to deliver care and patient health outcomes (Lim et al., 2019). Evidence has accumulated regarding hospital work environments across many countries. Although limited, some cross‐national studies have attempted to compare the organizational features of hospitals (Aiken et al., 2011, 2024), but have rarely focused on nurses' health. A comprehensive understanding of both organizational work environments and individual health status between countries can help identify strengths and opportunities.

While increased patient needs in hospitals require a sufficient number of nurses to care for patients, there is a global hospital nurse shortage that is worsening in light of the COVID‐19 pandemic (Haryanto, 2019; Lopez et al., 2022). Inadequate nurse staffing hinders quality nursing care and threatens patient health (Butler et al., 2019). Various government policies have been implemented in each country to ensure sufficient nurse staffing so nurses can spend adequate time caring for each hospital patient. While minimum nurse‐to‐patient ratios have been mandated since 1999 in acute care hospitals in California, the United States, the Korean government has continued to expand the nurse supply by increasing the number of nursing educational institutions and their graduates (Coffman et al., 2002; Han et al., 2020; Organisation for Economic Cooperation and Development (OECD), 2021). It has been difficult to retain nurses in Korea due to a lack of sufficient work environment improvements.

Although there has been an increase in the total number of nurses in Korea (70 nurses in 2016 to 85 in 2020 per 10,000 people), the number of nursing graduates per 100,000 people (100.2) should be more than double that of the OECD nations (44.5) to achieve a comparable number of practicing nurses per 1000 people (7.9 in South Korea vs. 8.8 in the OECD countries), suggesting a low nurse retention rate in Korea (OECD, 2021). The turnover rate of nurses within their first year of work was 26.4% in 2018–2019, compared to 22% in 2012–2016 (Kim & Kim, 2021; Park & Ko, 2020). This suggests a rising turnover rate despite the Korean government's policies to increase the number of nurses and reduce turnover (Kim et al., 2017).

Nurses in South Korea reportedly care for approximately 12 patients per shift, which is twice the number of patients cared for by nurses in the United States and in some European countries (Cho et al., 2015, 2016). A cross‐national study of nine countries reported high burnout and job dissatisfaction among Korean nurses (Aiken et al., 2011). Among work environment domains, Korean nurses rated staffing and resource adequacy as low. Korean nurses worked fewer years (5.8 years) compared to US nurses (15 years). The negative impact of such unfavourable occupational factors on workers' health and well‐being can potentially be mitigated through organizational employee health support efforts (Saeed et al., 2023). Korean nurses exhibit a lower level of health‐promoting behaviour practices, e.g. quality sleep, healthy diet behaviours and regular physical activity, compared to US nurses (Cho & Han, 2018; Lim et al., 2019). Korean nurses report more occupational stress and burnout compared to US nurses (Ha et al., 2021). Although nurses' health is recognized as an important factor for quality nursing care and should be thoroughly explored (Cho et al., 2018), cross‐national comparisons of organizational‐ and individual‐level health‐related characteristics have rarely been reported among nurses.

Cross‐national differences in the healthcare system and nursing workforce structure elucidate nurses' perception of their work and health statuses. Healthcare financing is notably different between South Korea and the United States, as South Korea offers universal healthcare coverage through the National Health Insurance programme, while the United States primarily has private health insurance (Kim & Jacobson, 2022). Nursing care in the United States is often delivered via interdisciplinary teams involving physicians, nurse practitioners, physician assistants and other healthcare professionals. Nurses in South Korea cover expanded roles and responsibilities typically taken by assistant personnel or physicians in the United States. Comparisons between countries can be one of the strategies to achieve quality care delivery, as it helps identify the strengths and opportunities that are specific to one location compared to another.

When comparing occupational features between cultures, inherent differences in demographic characteristics should be considered. For example, senior and male nurses tend to perceive work environments positively and be involved in health‐seeking behaviours compared to their younger, female counterparts (Baek et al., 2019; Cho et al., 2018). Without consideration of such demographic differences, it is not possible to determine whether the differences in work and health‐related perceptions among workers are attributable to potentially modifiable work‐related factors. To remove confounding effects and enhance between‐group comparability, analytic studies frequently match potential confounders (Yang et al., 2022).

In this study, we attempted to compare the work characteristics (work conditions, work schedules and organizational support and resources for employee health) and health (healthy behaviours and health status) between Korean and US hospital nurses.

## 2 METHODS

### 2.1 Design

This was a comparative secondary data analysis using cross‐sectional data from Korean and US hospital nurses.

### 2.2 Data sources and participants

For the Korean nurses, we used data from the 2020 Korean Hospital Nurses Health Behaviors and Health Status study (Korean Hospital Nurses Association, 2020), which were collected from May to July 2020 at the beginning of the COVID‐19 pandemic. Of the 355 hospitals with 100 or more beds located in South Korea, 63 were randomly selected by the proportional stratified sampling procedure based on the region (Seoul, other metropolitan areas, and provinces) and number of beds (100–399, 400–699, 700–799, and 1000 or above). The number of survey questionnaires per hospital was determined based on hospital bed size. The questionnaire packages were mailed to the hospitals and the paper‐based questionnaires were randomly distributed to units across hospitals. Nurses who voluntarily participated in the study provided their informed consent, completed the questionnaires, sealed them and returned them to a designated return box. Nurse Officers at each hospital collected the completed questionnaires and mailed them to the research staff. Of the 4175 questionnaires distributed to the 63 hospitals, 3499 questionnaires from 54 hospitals were returned, yielding return rates of 85.7% for hospitals and 83.8% for nurses.

For the US nurses, we used data from the Nurse Worklife and Wellness Study (NWWS), a mixed‐mode survey (online, mailed) conducted between November 2020 and February 2021. Details concerning the NWWS sampling and data collection procedures have been described elsewhere (Trinkoff et al., 2021). Briefly, 3973 randomly selected registered nurses (RNs) in nine states received recruitment notices via mail or email containing a web address for the online questionnaire. For those who did not respond to later reminder emails or postcards, a paper version of the NWWS questionnaire was sent. Finally, 1215 nurses provided responses (resulting in a 30.6% response rate).

For this study, as the Korean data were all from hospital nurses, we selected only hospital nurses for the US nurse data (*n* = 520). To construct a comparable nurse dataset, we matched nurse pairs on a one‐to‐one basis in the two countries by gender and age (21–25, 26–30, 31–35, 36–40, 41–45, 46–50, 51–55, 56–60, 61+ years). The finalized dataset contained 304 matched pairs.

### 2.3 Measures

To reduce participant response loading during the survey, increase the comparability of this cross‐national study, and support reliability and content validity, we selected items frequently used in work and wellness research (Hoeppner et al., 2011; Park & Johantgen, 2017).

We measured work conditions using three items concerning staffing adequacy, time pressure and salary adequacy via 4‐point Likert‐type response options (1 = “strongly disagree” to 4 = “strongly agree”) (Aiken et al., 2011; Baek & Trinkoff, 2022). Nurses were asked about their typical schedules over the past 6 months in terms of (1) shift type, (2) hours worked per day and week (including overtime), (3) weekends worked per month and (4) the number of breaks lasting 10 min or more, including meals. These items were extracted from the Standard Shiftwork Index, a self‐report instrument widely utilized in international shift‐work research (Barton et al., 1995; Cho et al., 2021). To capture nursing‐specific work schedule characteristics, four additional items were included: how often nurses worked (1) 1 h or more over time, (2) with less than 10 h off between shifts, (3) on a scheduled day off or a vacation day and (4) while sick. Their response options were 0 = ‘never’, 1 = ‘rarely’, 2 = ‘sometimes’ and 3 = ‘often’. Three experts from the National Institute for Occupational Safety and Health assessed the content validity of these variables (Trinkoff et al., 2006). Additionally, we assessed the work outcomes of job satisfaction, intention to stay at their current workplace, and the quality of nursing care using single items, each via 4‐point Likert‐type options (1 = ‘strongly disagree’ to = ‘strongly agree’).

Workplace wellness support availability was assessed for healthy food choices, wellness programmes and facilities, which are included in the US nationwide ‘Healthy Nurse Healthy Nation’ survey (American Nurses Association, 2024; Baek & Trinkoff, 2022). Korean nurses were asked about the availability of (1) healthy food choices, (2) wellness health promotion programmes and (3) employer‐based exercise facilities and programmes using 4‐point Likert‐type response options (1 = ‘strongly disagree’ to 4 = ‘strongly agree’). The US nurses were given four response options (‘don't know/not sure’, ‘not available’, ‘available but not used’, and ‘available and used’) for the four items on availabilities concerning (1) healthy food choices, (2) yoga programmes, (3) exercise space (e.g. gym) and (4) exercise classes/programmes.

Health behaviours were measured using three items each for physical activity (vigorous activity, light‐moderate activity and stretching or weight‐bearing) and a healthy diet (low in saturated fat, limited sugary foods/drinks, five fruits or vegetables/day). These items were sourced from the Health Promoting Lifestyle Profile‐II, which demonstrated established content, construct, and criterion validity, along with good reliability in this study (*α* = 0.71–0.84) (Cho & Han, 2018; Walker et al., 1996). For health status, we included three items concerning sleep (i.e. sleep quality, refreshing sleep and difficulty falling asleep) and a single item on self‐rated health status (Han et al., 2019). For the sleep quality item, the Korean nurse data were measured using 4‐point response options (1 = ‘very poor’, 2 = ‘poor’, 3 = ‘good’ and 4 = ‘very good’) versus the US data, which employed 5‐point responses (1 = ‘very poor’, 2 = ‘poor’, 3 = ‘fair’, 4 = ‘good’ and 5 = ‘very good’).

We also assessed general participant characteristics, including age, gender, educational attainment, years of RN experience and work positions.

### 2.4 Data analysis

The data were analysed using IBM SPSS Statistics for Windows, version 26 (IBM Corp., Armonk, NY, 2019). Descriptive statistics were presented as frequencies, percentages, means and standard deviations.

## 3 RESULTS

The participant characteristics of the original 2020 Korean Hospital Nurses Health Behaviors and Health Status study (*n* = 3499) and the US NWWS (*n* = 1215) were explored. In the Korean nurse dataset, the nurses were, on average, 32 years old with 8 years of RN experience; 95% were female; and 83% had a bachelor's degree or higher. While the respondents could not be compared with the national benchmark due to a lack of available national‐level research data, they were slightly older than those in previous large‐sized nurse samples (28 years old) (Cho et al., 2013, 2016). Regarding the US nurse data, age, gender and education in the NWWS were comparable and representative of the US RN workforce (Smiley et al., 2021). The median age was 52 years; 91% of the respondents were female; and 73% had a bachelor's degree or higher.

The matched samples of the nurses were an average of 39 years old with 13–14 years of RN experience. Approximately 92% of the respondents were female (Table 1). Nearly 72% of Korean nurses and 82% of US nurses had a bachelor's degree or higher. More than half of the nurses were staff nurses (62% of Korean nurses and 55% of US nurses).

**TABLE 1:** Participant characteristics of Korean and US hospital nurses.

	304 matched Korean hospital nurses				304 matched US hospital nurses				
	*n*	%	*M*	SD	*n*	%		*M*	SD
Age									
20–29	49	16.1	38.9	9.1	53	17.4	39.2	9.5	
30–39	93	30.6			95	31.3			
40–49	48	15.8			49	14.1			
50+									
Gender									
Female	281	92.4			281	92.4			
Male	23	7.6			23	7.6			
Educational attainment									
Associate	77	25.3			55	18.1			
BSN	166	54.6			184	60.5			
Masters or higher	54	17.8			64	21.1			
No response	7	2.3			1	0.3			
Years of RN experiences			14.1	8.6			13.3	9.4	
Position									
Staff nurses	190	62.5			168	55.3			
Charge nurses	100	32.9			81	26.6			
Others	12	3.9			53	17.4			
No response	2	0.7			2	0.7			

Abbreviations: M, mean; SD, standard deviation.

Overall, Korean nurses rated their job‐related conditions as much lower (e.g. staffing adequacy, time pressure and salary) than US nurses (Table 2). For example, only 27% of the Korean nurses versus 43% of the US nurses reported their hospital staffing levels to be adequate. The work schedule characteristics varied across the two country samples: in Korea, hospital nurses mostly worked rotating schedules (92% vs. 14% in the United States), and fixed night shift schedules were rare (0.3% in Korea vs. 21.7% in the United States). While hours worked per day were similar in Korea and the United States (9 h and 10 h, respectively), weekly work hours were longer in Korea (45 h) than in the United States (40 h). Furthermore, Korean nurses were less satisfied with their jobs and had lower intentions of staying in their current workplace than US nurses. Approximately 69% of Korean nurses and 94% of US nurses reported their quality of nursing care as good.

**TABLE 2:** Work‐related characteristics of Korean and US hospital nurses.

	304 matched Korean hospital nurses		304 matched US hospital nurses	
	*n*	%	*n*	%
Work conditions				
The staffing is adequate				
Strongly disagree	93	30.6	69	22.7
Disagree	125	41.1	102	33.6
Agree	80	26.3	104	34.2
Strongly agree	3	1	28	9.2
No response	3	1	1	0.3
(*M* ± SD)	(1.98 ± 0.79)		(2.30 ± 0.80)	
I have enough time to get the job done				
Strongly disagree	31	10.2	27	8.9
Disagree	118	38.8	78	25.7
Agree	145	47.7	168	55.3
Strongly agree	8	2.6	30	9.9
No response	2	0.7	1	0.3
(*M* ± SD)	(2.43 ± 0.71)		(2.67 ± 0.78)	
The salary is good				
Strongly disagree	64	21.1	17	5.6
Disagree	147	48.4	80	26.3
Agree	88	28.9	154	50.7
Strongly agree	2	0.7	51	16.8
No response	3	1	2	0.7
(*M* ± SD)	(2.09 ± 0.72)		(2.79 ± 0.75)	
Work schedules				
Shift type				
Permanent fixed schedule	20	6.6	223	73.3
Day only	19	6.3	149	49
Evening only	0	0	8	2.6
Night only	1	0.3	66	21.7
Rotating schedule	279	91.8	43	14.1
Others or missing	5	1.6	38	12.5
Hours worked per day, including overtime, *M* ± SD	9.03 ± 1.49		9.77 ± 2.57	
Hours worked per week, including overtime, *M* ± SD	45.01 ± 5.37		39.53 ± 13.85	
Weekends worked per month, *M* ± SD	2.06 ± 0.96		1.29 ± 1.34	
Number of breaks of 10+ minutes including meals during shift, M ± SD	1.27 ± 1.43		1.60 ± 1.06	
How often do you work with one hour or more over time?				
Never	50	16.6	109	36.6
Rarely	35	11.6	88	29.5
Sometimes	69	22.9	50	16.8
Often	147	48.9	51	17.1
(*M* ± SD)	(1.98 ± 0.93)		(1.14 ± 1.18)	
How often do you work with <10 h off between shifts?				
Never	168	56	128	43.8
Rarely	42	14	75	25.7
Sometimes	31	10.3	48	16.4
Often	59	19.7	41	14
(*M* ± SD)	(0.94 ± 1.16)		(1.01 ± 1.04)	
How often do you work on your day off/vacation day?				
Never	135	44.9	116	38.9
Rarely	129	42.9	88	29.5
Sometimes	27	9	69	23.2
Often	10	3.3	25	8.4
(*M* ± SD)	(0.71 ± 0.81)		(1.01 ± 0.98)	
How often do you work while sick				
Never	71	23.6	107	36.1
Rarely	168	55.8	98	33.1
Sometimes	48	16	74	25
Often	14	4.6	17	5.7
(*M* ± SD)	(1.02 ± 0.78)		(1.00 ± 1.08)	
Work outcomes				
I am very satisfied with my job				
Strongly disagree	32	10.5	14	4.6
Disagree	137	45.1	85	28
Agree	126	41.4	140	46.1
Strongly agree	7	2.3	63	20.7
No response	2	0.7	2	0.7
(*M* ± SD)	(2.36 ± 0.70)		(2.84 ± 0.87)	
I plan on staying at the current workplace for the next year				
Strongly disagree	25	8.2	19	6.3
Disagree	64	21.1	31	10.2
Agree	180	59.2	152	50
Strongly agree	31	10.2	99	32.6
No response	4	1.3	3	1
(*M* ± SD)	(2.72 ± 0.76)		(3.10 ± 1.07)	
The quality of nursing care is very good				
Strongly disagree	3	1	0	0
Disagree	92	30.3	16	5.3
Agree	196	64.5	183	60.2
Strongly agree	13	4.3	103	33.9
No response	–	–	2	0.7
(*M* ± SD)	(2.72 ± 0.56)		(3.29 ± 1.11)	

Abbreviation: *M* ± SD, mean ± standard deviation.

Korean nurses reported that organizational support and resources for employee health were less prevalent (e.g. available healthy foods and exercise opportunities) than US nurses (Table 3). Approximately 45% of the Korean nurses versus 78% of the US nurses reported healthy foods were available at their workplace, and 25% of Korean nurses versus 29%–42% of the US nurses reported that exercise facilities/programmes were available at their workplace. Healthy behaviour practices (e.g. physical activity, healthy diet) and health status (sleep quality and self‐rated health) were all rated lower by the Korean nurses than by those in the United States (Table 4).

**TABLE 3:** Workplace wellness support availability among Korean and US hospital nurses.

	304 matched Korean hospital nurses			304 matched US hospital nurses	
	*n*	%		*n*	%
Healthy food choices			Healthy food choices		
Strongly disagree	44	14.5	Don't know/not sure	6	2.0
Disagree	121	39.8	Not available	61	20.1
Agree	133	43.8	Available but not used	51	16.8
Strongly agree	5	1.6	Available and used	186	61.2
No response	1	0.3	No response	–	–
(*M* ± SD)	(2.32 ± 0. 63)				
Wellness health promotion programmes			Yoga programme		
Strongly disagree	94	30.9	Don't know/not sure	38	12.5
Disagree	140	46.1	Not available	20.1	66.1
Agree	65	21.4	Available but not used	51	16.8
Strongly agree	3	1.0	Available and used	13	4.3
No response	2	0.7	No response	1	0.3
(*M* ± SD)	(1.93 ± 0.78)				
Employer‐based exercise facilities or programmes			Exercise space (e.g., gym)		
Strongly disagree	104	34.2	Don't know/not sure	13	4.3
Disagree	124	40.8	Not available	164	53.9
Agree	71	23.4	Available but not used	89	29.3
Strongly agree	4	1.3	Available and used	38	12.5
No response	1	0.3	Exercise classes/ programmes		
(*M* ± SD)	(1.92 ± 0.77)		Don't know/not sure	26	8.6
			Not available	189	62.2
			Available but not used	69	22.7
			Available and used	19	6.3
			No response	1	0.3

Abbreviation: *M* ± SD, mean ± standard deviation.

**TABLE 4:** Health behaviours and health of Korean and US hospital nurses.

	304 matched Korean hospital nurses		304 matched US hospital nurses	
	*n*	%	*n*	%
Health behaviours				
Vigorous activity				
Never	157	51.6	150	49.3
Sometimes	89	29.3	89	29.3
Often	36	11.8	36	11.8
Routinely	20	6.6	29	9.5
No response	2	0.7		
(*M* ± SD)	(1.73 ± 0.91)		(2.48 ± 1.27)	
Light‐moderate activity				
Never	92	30.3	33	10.9
Sometimes	109	35.9	116	38.2
Often	73	24.0	104	34.2
Routinely	28	9.2	51	16.8
No response	2	0.7		
(*M* ± SD)	(2.12 ± 0.95)		(2.57 ± 0.93)	
Stretching or weight‐bearing				
Never	112	36.8	115	37.8
Sometimes	108	35.5	92	30.3
Often	59	19.4	58	19.1
Routinely	22	7.2	34	11.2
No response	3	1.0	5	1.6
(*M* ± SD)	(1.97 ± 0.93)		(2.04 ± 0.93)	
Low in saturated fat				
Never	66	21.7	18	5.9
Sometimes	118	38.8	128	42.1
Often	102	33.6	99	32.6
Routinely	16	5.3	57	18.8
No response	2	0.7	2	0.7
(*M* ± SD)	(2.23 ± 0.85)		(2.65 ± 0.92)	
Limited sugary foods/drinks				
Never	100	32.9	24	7.9
Sometimes	125	41.1	107	35.2
Often	54	17.8	99	32.6
Routinely	17	5.6	72	23.7
No response	8	2.6	2	0.7
(*M* ± SD)	(1.96 ± 0.87)		(2.73 ± 0.88)	
5 fruits or vegetables/day				
Never	66	21.7	42	13.8
Sometimes	142	46.7	123	40.5
Often	75	24.7	88	28.9
Routinely	20	6.6	49	16.1
No response	1	0.3	2	0.7
(*M* ± SD)	(2.16 ± 0.84)		(2.48 ± 0.85)	
Sleep				
Sleep quality (in the past week)				
Very good	4	1.3	9	3.0
Good	130	42.8	100	32.9
Fair			143	47.0
Poor	156	51.3	44	14.5
Very poor	11	3.6	7	2.3
No response	3	1.0	1	0.3
(*M* ± SD)	(2.24 ± 0.82, range 1–4)		(3.20 ± 1.22, range 1–5)	
My sleep is refreshing				
Never	21	6.9	11	3.6
Rarely	115	37.9	70	23.0
Sometimes	132	43.5	130	42.8
Often	30	9.9	75	24.7
Always	5	1.6	17	5.6
No response	1	0.3	1	0.3
(*M* ± SD)	(2.61 ± 1.16)		(3.06 ± 1.20)	
I have difficulty falling asleep				
Never	30	9.9	44	14.5
Rarely (1–2 times a week)	100	32.9	91	29.9
Sometimes (3–4 times a week)	97	31.9	86	28.3
Often (5–6 times a week)	62	20.4	66	21.7
Always	12	39.4	17	5.6
No response	3	1.0		
(*M* ± SD)	(2.54 ± 1.16)		(2.74 ± 1.16)	
Self‐rated health status				
Very good	4	1.3	29	9.5
Good	84	27.6	147	48.4
Fair	182	59.9	103	33.9
Poor	31	10.2	21	6.9
Very poor	1	0.3	2	0.7
No response	2	0.7	2	0.7
(*M* ± SD)	(3.19 ± 1.23)		(3.60 ± 1.21)	

Abbreviation: *M* ± SD, mean ± standard deviation.

## 4 DISCUSSION

A comprehensive understanding of nursing work conditions and health status is critical for quality care delivery, which can be achieved by identifying improvement opportunities through a comparative analysis between countries. This study is the first to compare hospital nurses' work‐ and health‐related characteristics and workplace support for employees between the Korean and the US nursing contexts. Overall, Korean nurses rated their job‐related conditions worse, had a lower intention to stay in their current workplace and were less satisfied with their jobs than US nurses. Furthermore, Korean nurses reported that organizational support and resources for employee health were less prevalent and their healthy behaviour practices and health status were lower compared to their US counterparts. Given the insufficient organizational support for nurses' practices and health in Korea, policymakers and other stakeholders should continue to scrutinize this issue.

Consistent with our findings, hospital work environments, burnout, job satisfaction and quality of care outcomes are lower among Korean nurses than those in other countries (Ha et al., 2021; Jun et al., 2021). Korean nurses have a far higher nurse‐to‐patient ratio, as they care for approximately 11 patients per shift, which is twice that of nurses in the United States and some European countries. They are also exposed to greater work demands (Cho et al., 2015). Nurse retention and quality patient care cannot be achieved without improving the practice environment. The Korean government should take corrective actions, such as thoroughly defining and mandating adequate staffing levels.

Currently, the Korean government reimburses nursing management fees (i.e. Differentiated Inpatient Nursing Fees) to hospitals based on nurse staffing, with 6 grades for tertiary hospitals and 7 grades for general hospitals and hospital‐level institutions. The nurse‐to‐patient ratio without penalty ranges from 4.0:1 or more for tertiary hospitals and 4.5:1 and 6.0:1 for general hospitals. This indicates that one nurse is in charge of approximately 20 patients in tertiary referral hospitals, 22 patients in general hospitals and 29 patients in hospital‐level institutions. This is unreasonable compared with foreign standards (Shin et al., 2020). Although the inpatient nursing management fees are reimbursed depending on the hospital nurse staffing level, the actual staffing costs exceed these expenses. Furthermore, when hospitals' nurse staffing levels are at the lowest grade, the management fee is reduced by only 5%, which is far less than the nurse recruitment cost (Shin et al., 2020). Although nurses are essential to ensuring safe and quality patient care, they are seen as a cost centre (Lasater et al., 2021). To secure sufficient nurse staffing levels and provide favourable work conditions, the Korean government should impose considerable penalties on hospitals with suboptimal nurse staffing levels. The organizational context of hospital care delivery should be improved for better nursing and patient outcomes.

The health and well‐being of nurses is a critical concern given the COVID‐19 pandemic, in which many nurses have experienced greater work demands and more extensive hours than ever before. Organizational support and resources for employee health can enhance work performance, decrease costs and contribute to improved engagement and productivity (Attridge et al., 2021; Nunes et al., 2018). During the pandemic, institutional‐ and national‐level support effectively reduced the intention to leave of healthcare workers, who were at the forefront of the fight against the epidemic (Çetin Aslan, Türkmen, & Top, 2022). Specifically for Korean nurses, in addition to the lack of support, rotating schedules with night shifts are another factor hindering their health practices and adversely affecting their health status (Chang & Peng, 2021). A favourable organizational climate for nurses' health should be created for both countries. Hospitals should increase access to healthy food and exercise facilities/programmes for healthcare workers with non‐standard hours. To restrict unhealthful scheduling and to promote nurses' health, governments should regulate total work hours and mandatory overtime. For example, weekly work hour caps can be set to 40–48 h, including any overtime, which aligns with the European Union Working Time Directive (European Commission, 2003). To prevent fatigue and ensure nurses are well‐rested, nurses should be provided mandatory periods of at least 11 consecutive hours of rest between shifts and a minimum of 24 h of rest per week. In New York, US, all instances of mandatory overtime must be reported to the New York State Department of Labor to prohibit hospitals from forcing nurses to work overtime, except during limited emergency situations (The Laws of New York, 2023).

Our study's findings should be interpreted in consideration of the following limitations. First, our study sample might not be representative of all Korean and US nurses. As we matched nurses by age and gender, the Korean nurses in this study were, on average, older than the benchmark (39 vs. 31 years old). However, given that senior nurses reportedly practice healthy behaviours more frequently and perceive their work more positively than junior nurses (Baek et al., 2019; Lim et al., 2019), our study may underestimate the work and health‐related perceptions of the overall Korean nurse population. Second, whereas the US nurse data were collected through online or postal surveys (mailed to their homes), the Korean nurses received the survey questionnaire package at their workplaces and, therefore, would be more vulnerable to denial or deception of problems and social desirability. However, the Korean nurses were asked to seal the surveys immediately after completing the questionnaires and place them in a secure box. This ensured confidentiality and minimized bias. Finally, our study was based on single‐item comparisons, which are vulnerable to construct validity. However, single‐item comparisons are often employed in cross‐cultural comparison studies due to a lack of measurement instruments applicable to different settings (Hoeppner et al., 2011; Park & Johantgen, 2017). Future studies are warranted to include sound measurement tools.

## 5 CONCLUSIONS

Continued examination of the health system characteristics between Korea and the United States would help draw policy recommendations for nurses' quality care practices and good health. Our comparative study suggests that comprehensive approaches to improve practice environments and employee health support are required for Korean nurses compared to their US colleagues. In addition to such organizational efforts to improve health and work outcomes, organizational resources are recognized as a redeeming feature of job demands and further improve work and health outcomes (Saeed et al., 2023). Specifically, workplaces with high nursing workloads and demands should offer nursing health and wellness opportunities at the facility level (Grinspun, 2008). These workplace offerings should be designed to be accessible to nurses with irregular schedules or inadequate amounts of free time to pursue them, such as facility‐level health practices during breaks. Future cross‐national studies should employ random sampling in a similar manner to construct more comparative and generalizable samples and evaluate how the organizational features of hospital work environments impact nurse and patient outcomes.

## 6 IMPLICATIONS

This cross‐national study revealed that job‐related conditions were worse among Korean nurses compared to US nurses. The findings highlight opportunities for nurse leaders and managers to improve the organizational context and support for Korean nurses. To advance practice environments, nursing workload and adequate staffing levels should be thoroughly assessed. The Korean government should mandate sufficient nurse staffing levels in hospitals for safe and healthy care practices. To provide organizational support and resources for employee health, hospitals should offer accessible healthy food and exercise facilities/programmes for healthcare workers around the clock. Accessible psychological support services that provide career counselling and offer staff development opportunities may mitigate the desire to leave work and improve job satisfaction among nurses (Byrne et al., 2023; David et al., 2022). Finally, health system characteristics and workers' health status should be periodically evaluated to advance nurses' quality care practices.

## AUTHOR CONTRIBUTIONS

**Kihye Han**: Conceptualization, methodology, formal analysis, investigation, resources, data curation, writing–review and editing, project administration and supervision. **Alison M. Trinkoff**: Conceptualization, methodology, investigation, resources, data curation, writing–original draft, writing–review and editing. **Hyang Baek**: Conceptualization, methodology, investigation, data curation, writing–review and editing. **Yeonhee Kim**: Conceptualization, methodology, investigation, writing–review and editing.

## FUNDING INFORMATION

This study was supported by the Chung‐Ang University Research Grants in 2021, the Korean Hospital Nurse Association in 2020 and the US National Council State Boards of Nursing CRE Grant (#R19B07).

## CONFLICT OF INTEREST STATEMENT

The authors declare that there is no conflict of interests.

## ETHICS STATEMENT

This study was approved by the Institutional Review Boards at the Chung‐Ang University (IRB no. 1041078‐202,203‐HR‐111) before study initiation.

## Data Availability

Data available on request due to privacy/ethical restrictions.
